# Supplementary material for: Inducible overexpression of a FAM3C/ILEI transgene has pleiotropic effects with shortened life span, liver fibrosis and anemia in mice
Source: PLoS One. 2023 Sep 15;18(9):e0286256. doi: 10.1371/journal.pone.0286256 (PMC10503705; doi:10.1371/journal.pone.0286256)
Supplement: S1 File — (ZIP) [file pone.0286256.s001.zip › PONE Figure 1/1D/KH2-ILEI-ESclones Dox WB.pptx]

## Slide 1
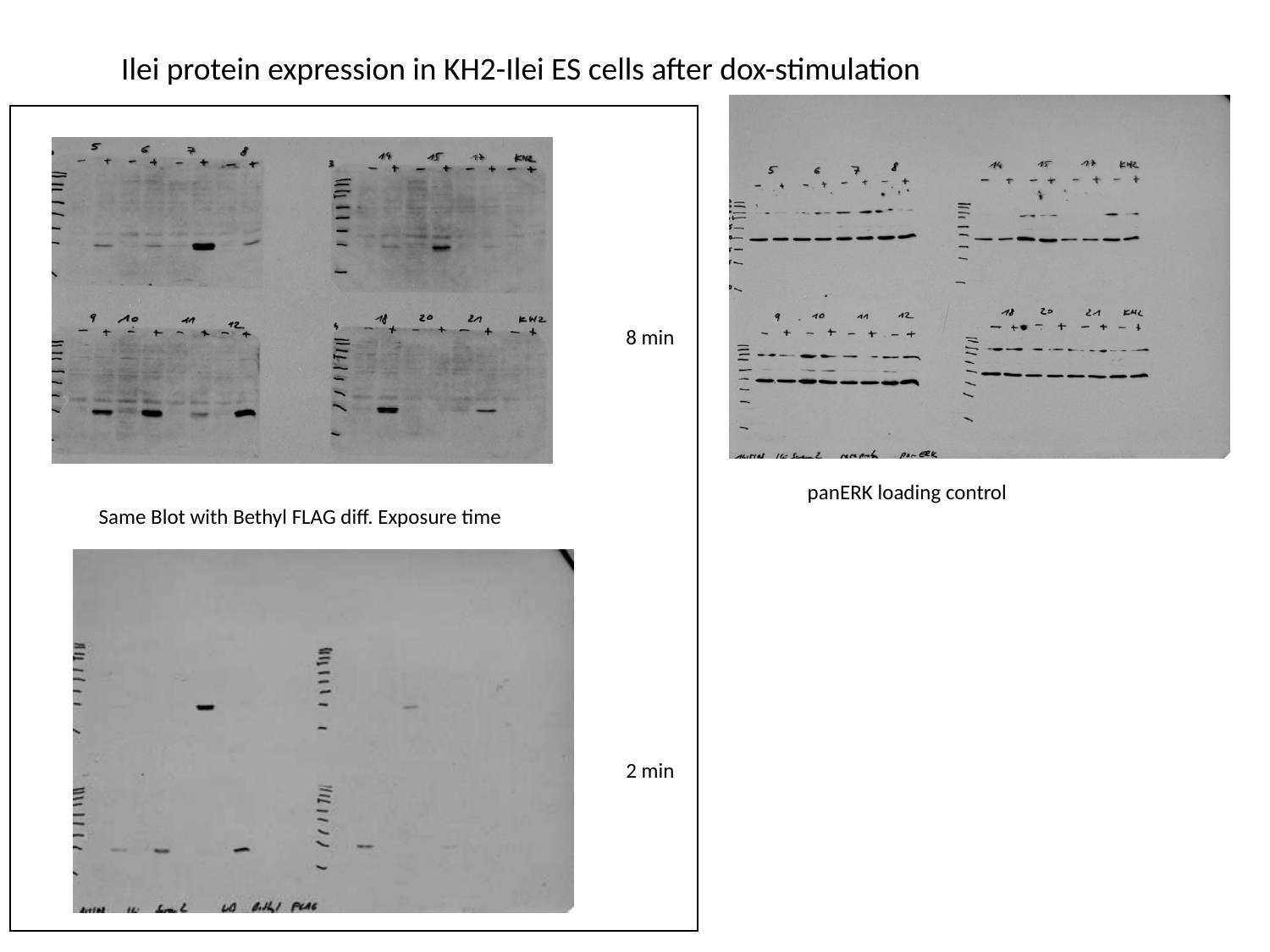

Ilei protein expression in KH2-Ilei ES cells after dox-stimulation
8 min
panERK loading control
Same Blot with Bethyl FLAG diff. Exposure time
2 min
