# Supplementary figures and images for: Inducible overexpression of a FAM3C/ILEI transgene has pleiotropic effects with shortened life span, liver fibrosis and anemia in mice
Source: PLoS One. 2023 Sep 15;18(9):e0286256. doi: 10.1371/journal.pone.0286256 (PMC10503705; doi:10.1371/journal.pone.0286256)

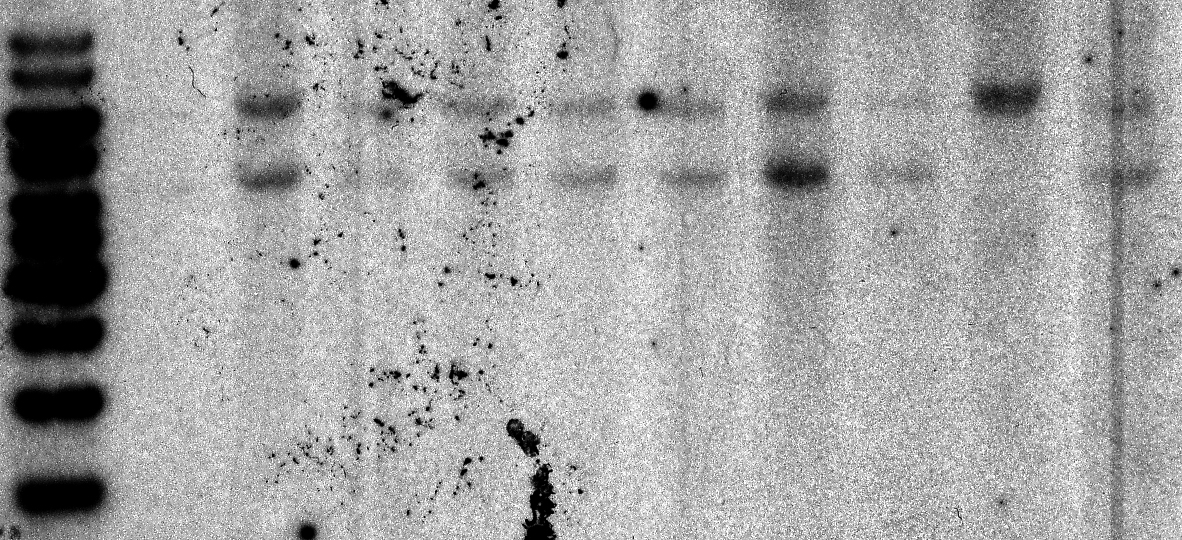

Supplement: S1 File — (ZIP) [file pone.0286256.s001.zip › PONE Figure 1/1E/KH2-ILEI Southern.jpg]

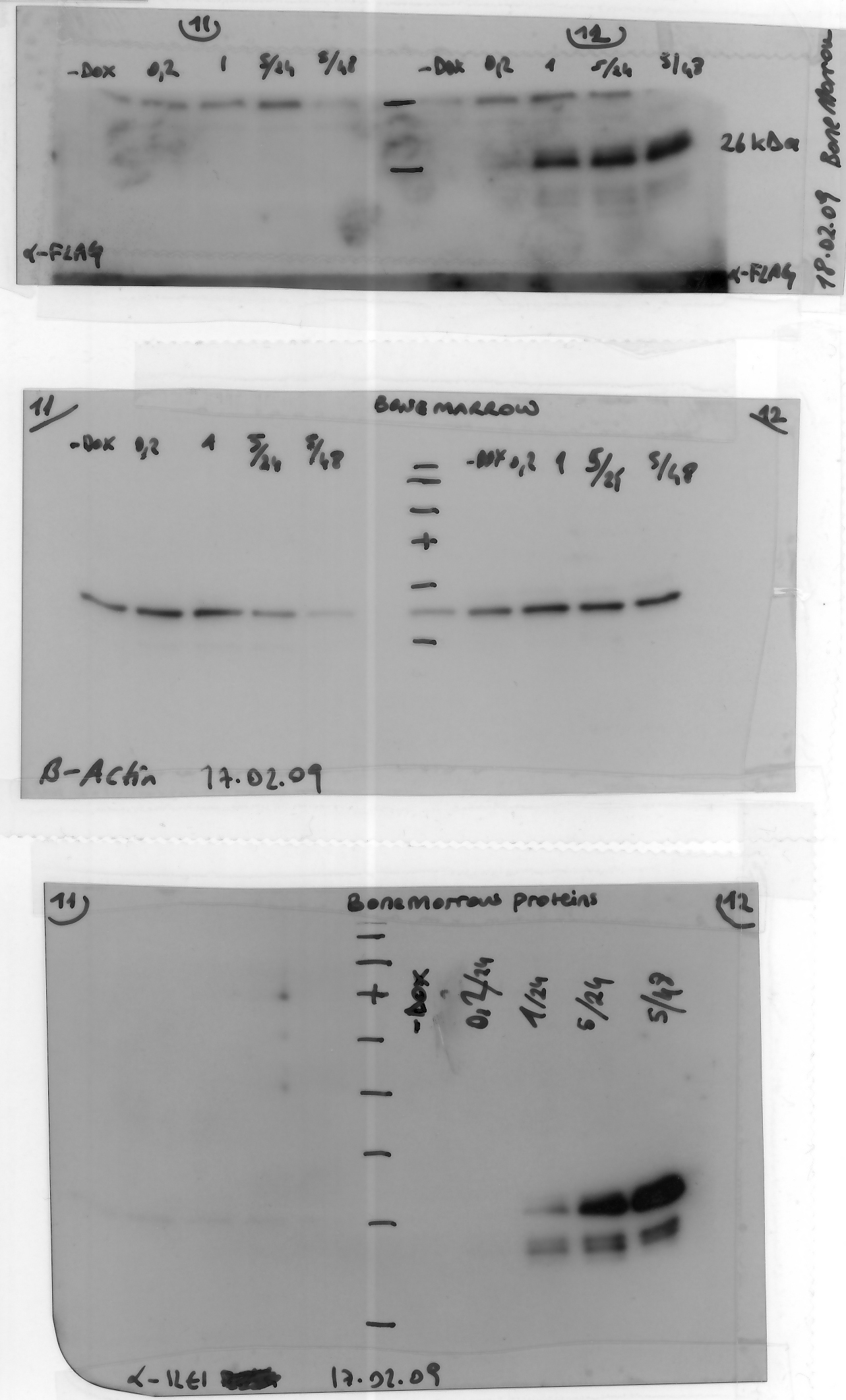

Supplement: S1 File — (ZIP) [file pone.0286256.s001.zip › PONE Figure 2/2A/uncropped_exvivo bone marrow Dox inductionWestern.jpg]

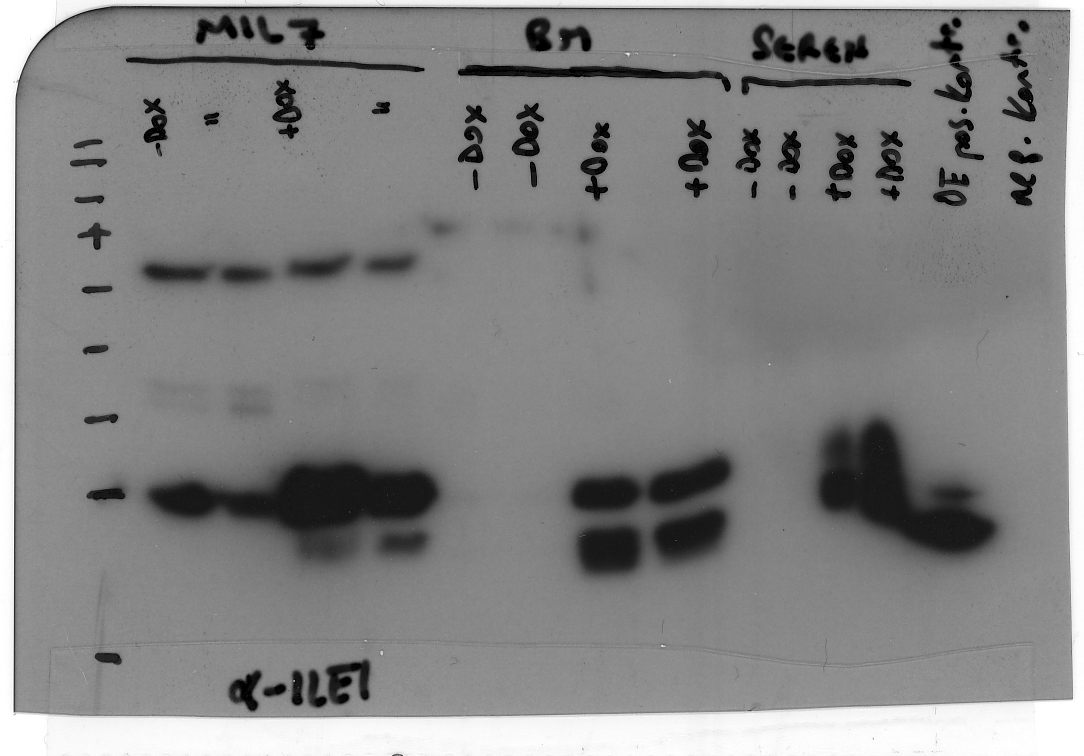

Supplement: S1 File — (ZIP) [file pone.0286256.s001.zip › PONE Figure 2/2B/in vivo Dox_spleen_BM_serum ILEI.jpg]

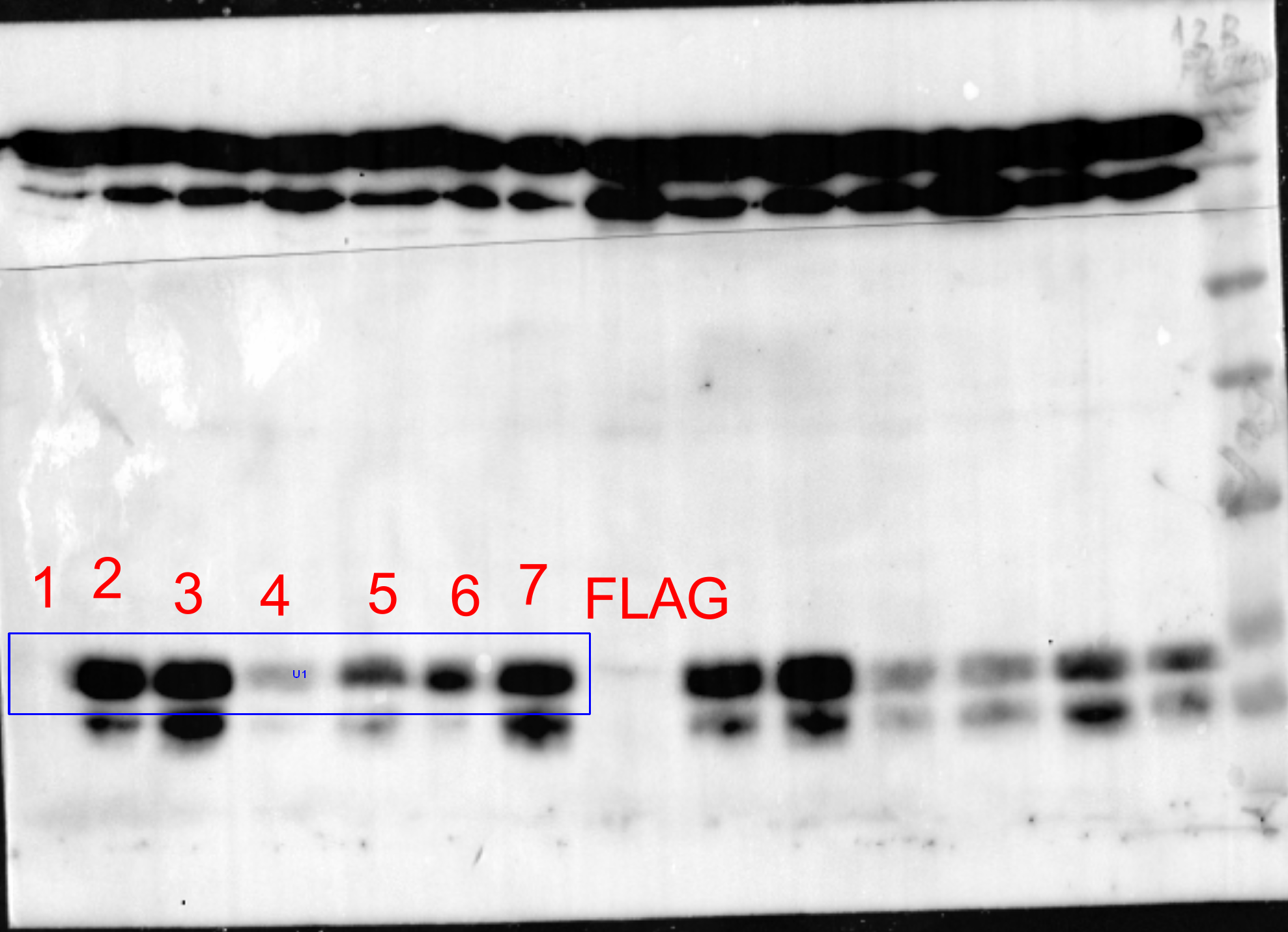

Supplement: S1 File — (ZIP) [file pone.0286256.s001.zip › PONE Figure 5/5A/uncropped FLAG_MWmerge.tif]

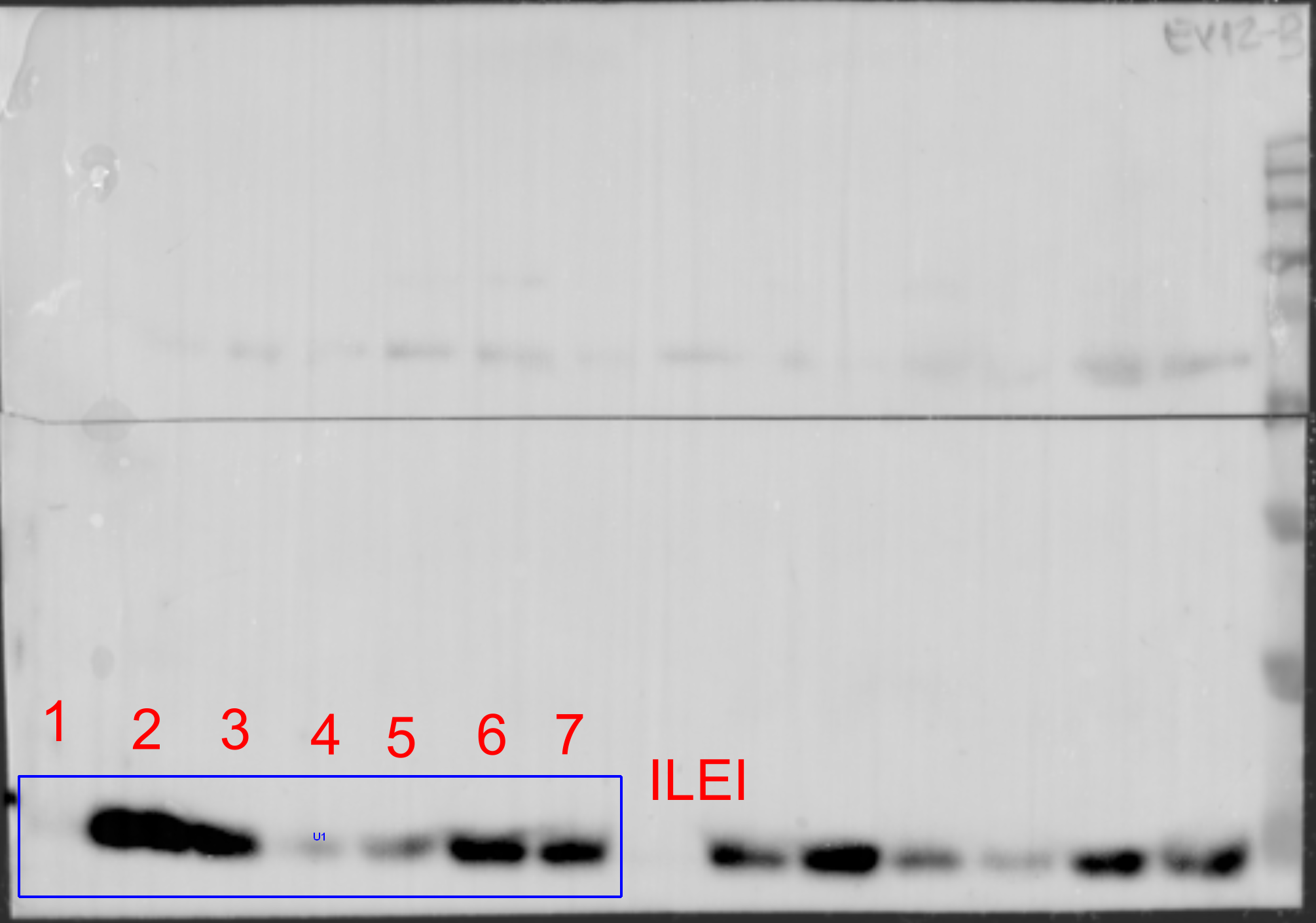

Supplement: S1 File — (ZIP) [file pone.0286256.s001.zip › PONE Figure 5/5A/uncropped ILEI_MWmerge.tif]

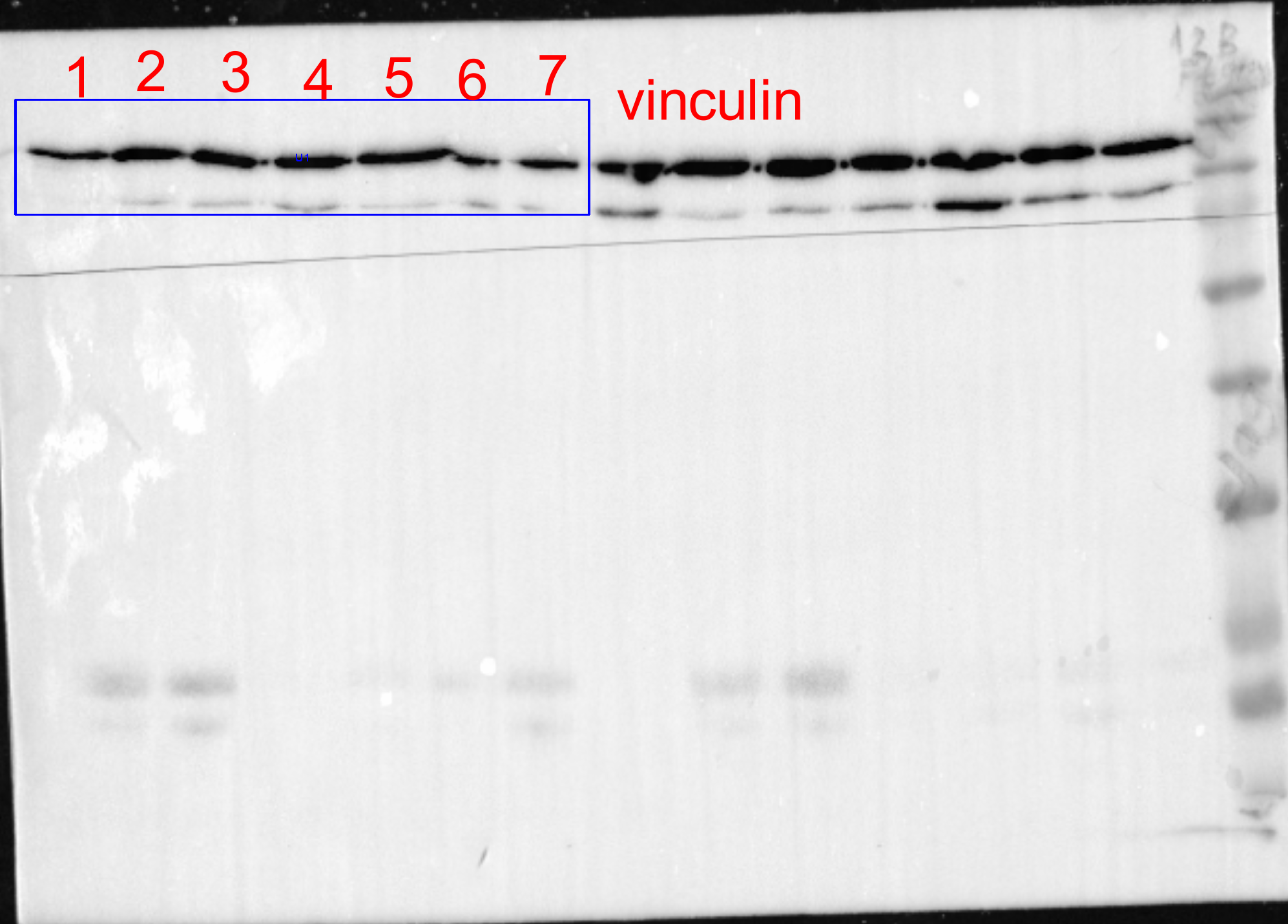

Supplement: S1 File — (ZIP) [file pone.0286256.s001.zip › PONE Figure 5/5A/uncropped vinculin_MWmerge.tif]
