# Supplementary material for: Inducible overexpression of a FAM3C/ILEI transgene has pleiotropic effects with shortened life span, liver fibrosis and anemia in mice
Source: PLoS One. 2023 Sep 15;18(9):e0286256. doi: 10.1371/journal.pone.0286256 (PMC10503705; doi:10.1371/journal.pone.0286256)
Supplement: Attachment — Submitted filename: Response to Reviewers.docx [file pone.0286256.s002.docx]

**Reviewers' comments:**

**5. Review Comments to the Author**

***Reviewer #1****: The manuscript PONE_D_23-00773 by Schmidt et al. is very interesting indicating tissue-specific effects for ILEI overexpression. The manuscript is well written and the data presented support the conclusions.*

We are very thankful to the Reviewer for his/her supportive words and appreciate his/her interest in our findings.

***Reviewer #2****: In this study, the authors generated and characterized a Tet-ON inducible ILEI-transgenic mouse strain. They found that mice with induced ubiquitous ILEI-FLAG overexpression at weaning age exhibited a shortened lifespan, reduced body weight, iron-deficiency anemia, and liver fibrosis with dysfunction. Reduced iron levels were observed in the serum and in hepatocytes. This study is an initial and incomplete characterization of Tet-ON inducible ILEI-transgenic mice. This reviewer has the following comments on this manuscript:
1) Using quantitative methods such as immunoblotting and ELISA, the authors should show expression levels of ILEI in major organs including the brain, heart, lung, liver, spleen, kidney, skeletal muscles, and bone marrow of induced ILEI-Tg mice used in this study as comparing with those of control and wild-type mice.*

We would like to thank the Reviewer for taking the time and effort to review our manuscript and for their suggestions in improving it. The Reviewer’s request on expression of ILEI in major organs was well taken. Originally, we focused on showing ILEI expression levels of the main organs that were relevant for the rest of the study: intestine, liver, and blood system (consisting of bone marrow, spleen, and serum). For the latter we indeed used immunoblotting as shown in **Figure 2B** and as the Reviewer suggests this allows visualization of the increased expression levels on induced expression. In the revised version, we have also extended the panel of IHC analysis to other organs including the lung and skin as part of **Figure 2C**. Unfortunately, we are unable to provide results for all of the organs suggested without repeating the experiments, which we think would be hard to justify in terms of the ethical use of animals. While we agree that the Reviewer’s suggestion for Western blotting would provide more quantitative information about the overall protein levels, we believe that the provided (and for revision extended) panel of IHC has the advantage of capturing expression patterns in the context of the tissue. The concomitant anti-FLAG IHC against the epitope-tag of the transgene ensures the specific recognition of transgene expression. The additional results highlighted subtle differences in expression patterns supporting the need for further experiments in future to fully evaluate the whole animal expression of ILEI. Accordingly, we have added the following to the results on page 13: “Of note, transgene expression was not detectable in the single-layer alveolar epithelium of the lung, but in other lung structures.” We have also updated the discussion on page 22 as follows: “This will obviously be an interesting study in the future alongside a detailed examination of other important organs such as the heart, skeletal muscles, and brain.”

As the Reviewer indicates, ELISA would be a valuable tool for the accurate quantification of expression levels. The Reviewer formulated our dreams to have purchasable ELISA kits for the quantification of murine ILEI. Unfortunately, this is currently not the case. There are a few reports on the use of ILEI ELISA for the quantification of the human protein, but also these reports did not provide information on the manufacturer. There is a single report on ELISA-based mouse ILEI quantification, and this scientific report is basically about the development of an ILEI sandwich ELISA (Nakano et al., *Journal of Alzheimer's disease*, 2021) indicating that those authors also missed purchasable validated resources. We hope that increasing visibility and interest in ILEI research, e.g. through studies like ours, will also facilitate the commercial development of resources required for such studies.

*2) Previously, the author's group reported that homodimers of ILEI are exclusively active (Krai M, 2017; Jansson AM, 2017). They should examine whether overexpressed ILEI-FLAG in their mice is dimerized.*

We thank the Reviewer for highlighting that our dimerization studies are so well recognized. While we agree that this would add interesting information to the study, the technical challenges involved make it very difficult to present publication-quality results and may not provide a straightforward message. First, it is extremely difficult to reliably detect ILEI dimers in tissues, I myself would give the harshest critics to such Western blots, as we tested many different organs and know the difficulties in identifying the very weak specific signal from the background. The vast majority of our studies were restricted to in vitro cultures. Unfortunately, there are still no tools for the specific detection of ILEI dimers, the only way is through non-reducing Western blots. Second, more importantly, ILEI dimers always co-exist with ILEI monomers, at least this is what we have observed so far in every condition we analyzed, indicating that there is a dynamic balance between the two forms. Therefore, even if dimers can be detected in these mice, this would not provide us with any conclusion on the functionality and importance of dimerization, as one would not know to which form the observed phenotype should be linked. We could only adequately address this question by generation of dimerization-disabled ILEI transgenic or knock-in mouse strains. This is certainly something to consider for detailed study in the future as these mice would be enormously interesting to analyze, as we reported in our dimerization studies, we found some ILEI functions in vitro that were linked to ILEI monomers, e.g. cellular motility. However, we hope the Reviewer agrees that this is beyond the current scope of this study.

*3) Iron deficiency anemia is usually microcytic hypochromic but not normocytic hypochromic. The authors should show MCV and MCH (or MCHC) values. In addition, effectiveness to iron administration can indicate whether anemia of ILEI-overexpressed mice is caused by iron deficiency.*

We thank the Reviewer for raising this important point. We measured these values during our analysis and evaluated the results at submission for the temporary Dox induction in young mice. Those data showed reduced MCHC without reduced MCV, therefore our initial conclusion was normocytic anemia. However, upon the Reviewer’s well justifiable concerns, we also undertook the analysis of these values in moribund older mice and their age-matched controls. This analysis revealed that the Reviewers anticipation was true, in this setting alongside reduced MCHC, reduced MCV was apparent. This provided a clear case for microcytic hypochromic anemia, rather than normocytic as we previously thought. We have most probably misinterpreted the data in young mice due to interfering effects by the changes in erythropoiesis during postnatal development up to adulthood. We apologize for this apparent mistake and thank the Reviewer again for guiding us to recognize this mistake. By this we were also able to establish a more coherent link between the observed anemia and iron deficiency. We have now provided all MCV, MCH and MCHC values for both sets of experiments in the revised manuscript. Due to the increased amount of data, we have now split Figure 3 into two parts. The new Figure 4 consists of the temporary Dox induction study and Figure 3 shows the observations from continuous Dox supply.

In the Results following sentences were added:

1. For the anemia of moribund mice on page 15: “These reduced parameters were accompanied with reduced mean corpuscular volume (MCV), mean corpuscular hemoglobin (MCH), and mean corpuscular hemoglobin concentration (MCHC) values, indicative of microcytic hypochromic anemia.”

2. For the temporarily induced anemia in young mice on page 16: “accompanied with significantly reduced MCHC levels, whereas MCV reduction, a sign of microcytic anemia, was not consistent, most likely having been masked by the interfering size reduction of erythrocytes during postnatal development until adulthood (38).”

In the discussion on page 22 we explained: “Iron deficiency anemia normally presents as microcytic hypochromic anemia where RBCs have a smaller mean corpuscular volume (MCV) than normal. In accordance with this, mice in this study had also reduced MCV, indicative of microcytic hypochromic anemia caused by iron deficiency.”

The effect of iron administration studies would be a completely new set of animal experiments of several months, as first the anemia had to be established and then the monitoring phase of the “iron therapy”. We hope the Reviewer agrees that these experiments go beyond the focus of this study. Due to the pathological liver conditions, we can anticipate that anemia is linked to liver disfunction and not purely to iron deficiency, by this the additive value would be moderate compared to the high number of additional animals and experimental resources required for a detailed study.

Reference:

38.	Chen L, Wang J, Liu J, Wang H, Hillyer CD, Blanc L, et al. Dynamic changes in murine erythropoiesis from birth to adulthood: implications for the study of murine models of anemia. Blood Adv. 2021;5(1):16-25.

*4) Transferrin plays a critical role in transfer and metabolism of iron. The authors should measure serum transferrin levels of mice with induced ILEI overexpression.*

We agree with the Reviewer, there are many parameters one can measure to get a more precise picture on the nature of the anemic phenotype in these mice, including analyzing transferrin levels in the blood. Unfortunately, we had difficulties in collecting high blood volumes from the overexpressing mice by the time they started to show weakening fitness and became moribund. The collected samples were used up in performing the measurements that are in the manuscript, including blood test, flow cytometry and specifically from the serum: AST, ALT, and serum iron determination. Because of this, we do not have matched serum left to perform this measurement. For such measurements we would need to breed new animals, wait until they reach moribund age, perform blood collection with the hope to collect enough to be able to determine transferrin levels together with confirming measurements on anemia and serum iron levels. In addition, we are also not convinced that any measured values could have a clear conclusion. On the one hand, due to reduced iron in the serum we could expect increased transferrin levels, as iron deficiency has been described to induce elevated transferrin expression. On the other hand, however, we cannot exclude that liver dysfunction would lead to disturbed transferrin levels, as liver is the main producer of transferrin. By this, we are also not convinced that this additional measurement would be very conclusive and would largely contribute to a better understanding of the phenotype. Therefore, to partly address this point we have updated the discussion on page 24 to expand our plans for further studies as follows “Therefore, the mechanisms behind iron malabsorption indicative in this mouse model need to be investigated in more detail in the future. Those studies will involve uncoupling the effects of overexpression of ILEI in the liver from other systemic effects on iron levels and will require detailed analysis of various tissue specific ILEI expression profiles, including the intestine as main place of iron absorption. It is also important to fully understand the microcytic hypochromic anemia discovered in the mice, including analysis of the factors involved, such as transferrin, transferrin saturation and ferritin, to give a more precise understanding of its nature.”

*5) Livers of induced ILEI Tg mice were reduced in size, showed increased apoptosis, reduced cellular iron content, and had a fibrotic phenotype. Which finding appeared firstly? Were these findings reversible upon withdrawal of ILEI induction?*

Indeed, this is a very interesting point and would help reveal much about the mechanisms involved in liver dysfunction in the study. First, we have to state that we were not able to link all these phenotypes between the two sets of experiments. Temporary ILEI induction was terminated after 10 weeks, in mice aged 13 weeks (91 days). At this time point we did not sacrifice any animals to analyze the liver phenotype in detail histologically, rather, we wanted to see if the anemia was reversible, thus we let these mice to recover for another 4 weeks without doxycycline administration. At the time point of sacrifice we did not observe pathological liver histology, but we cannot tell if this was due to reversion or if the liver stayed intact. In these mice we did not measure serum levels of AST and ALT, as we observed pathological change of these values only 1-2 weeks before death. The earliest death event under continuous induction we recorded at 144 days of age, thus, another 7-8 weeks later. Even at 240 days we observed only 60% penetrance of the phenotype. As none of the mice that were terminated at endpoint without a moribund phenotype showed signs of liver dysfunction nor anemia, and it was not predictable in younger mice, which ones would develop these phenotypes later, we were not able to gradually monitor and map the sequence of pathological events. As the first signs we observed were anemia, and at this point it was rare to have pathological levels of AST and ALT, the serum markers of liver disfunction, we conclude that anemia might have appeared earlier, but we did not analyze livers when anemia was first observed in these older mice. We also did not perform any histological analysis of livers before the anemia phenotype, as we had no means to select/predict, which mice will develop later an anemic phenotype. Thus, we were not able to address really the question if anemia was a consequence of liver disfunction or happened already as primary event before liver pathology. Ethically and practically we were not able to follow up blood parameters from weaning age up to potential death at 20-35 weeks of age by weekly bleedings. Nevertheless, the observation that young mice showed anemia with 100% penetrance, while older mice had only 40-60% indicates that a subset of mice might have been able to compensate for anemia and potential initial liver malfunctions driven by ILEI overexpression as they aged. As the unpredictable timely appearance and partial penetrance of the phenotype had requested the histological analysis of much higher number of mice at many more intermediate time points, we do not feel conclusive enough to discuss these points in the manuscript, but we provided here a summary of our current understanding to the Reviewer. In the discussion on page 24 we suggest that “overexpression of ILEI in the liver results in liver dysfunction and as a consequence iron metabolism is disturbed”, this has now been expanded as “However, it is also possible that low iron levels are primary and the two phenotypes are initially not tightly linked.” We also highlighted the need to fully study the “Detailed analysis of this likely complicated mechanism is beyond the initial characterization of the mouse model presented here. Therefore, the mechanisms behind iron malabsorption indicative in this mouse model need to be investigated in more detail in the future. Those studies will involve uncoupling the effects of overexpression of ILEI in the liver from other systemic effects on iron levels and will require detailed analysis of various tissue specific ILEI expression profiles, including the intestine as main place of iron absorption.”

*6) Livers of mice with induced ILEI overexpression were somewhat reminiscent of liver cirrhosis in human. The authors should discuss about similarity and difference between these conditions.*

We agree with the Reviewer that this is an interesting point and worthy of discussion. So, we have added the following section to the discussion on page 22 to describe the similarities and differences of the mouse livers with cirrhosis: “The livers of mice overexpressing ILEI have many similar characteristics to liver cirrhosis in human disease, with evidence of liver dysfunction alongside increased levels of fibrosis markers and fibrous and disordered collagen. Cirrhosis is an advanced stage of liver fibrosis accompanied by distortion of the hepatic vasculature, which results in compromised liver function (46). Late-stage cirrhosis often results in smaller liver volume, as seen in this study, and this may be related to poor prognosis of patients with hepatocellular disease (47). On the other hand, cirrhosis usually develops after a sustained period of inflammation (46). In this study there was no evidence of a period of inflammation or hepatitis from ILEI overexpression.”

References:

46.	Ginès P, Krag A, Abraldes JG, Solà E, Fabrellas N, Kamath PS. Liver cirrhosis. Lancet. 2021;398(10308):1359-1376.

47.	Hagan MT, Sayuk GS, Lisker-Melman M, Korenblat K M, Kerr TA, Chapman WC, et al. Liver volume in the cirrhotic patient: does size matter?. Dig Dis Sci. 2014;59(4):886-891.

*7) Previous studies suggested that ILEI overexpression caused carcinogenesis. The authors should evaluate frequency of spontaneous cancer development after induction of ILEI overexpression.*

The Reviewer is correct, earlier findings on ILEI might let us anticipate the probability of spontaneous tumor formation. We indeed checked this. We did not observe any cancer formation, neither in moribund animals at the timepoint of death, nor in surviving animals at 240 days that was set as experimental endpoint. To make sure this result is clearly stated, we have now included the following sentences in the Materials & Methods at the end of the “mice” chapter on page 8:

“Animals were checked daily to monitor their health status by assessing nesting performance and examining groomed fur and posture. Autopsy was performed on rare moribund mice and on all surviving animals at day 240 of life, which was set as the endpoint of the experiment. During macroscopic post-mortem examinations, we did not detect any spontaneous cancer formation.”

In addition, we added the following sentence on this issue into the Discussion on pages 23/24:

“Similarly, while cirrhosis increases the risk of liver cancer (45), we did not observe any sign of malignant transformation in the liver. Overall, ILEI overexpression did not cause spontaneous tumor formation in any organs as analyzed by autopsy of moribund and endpoint mice.”

*8) In line 370 on page 17; "Figure F" ?*

We thank the Reviewer for spotting this error. It should have read Figure 5F and has now been corrected as Figure 6F to reflect the new figure numbers.

*9) The authors refer to the same mouse line as "Fam3c transgenic" and "ILEI transgenic".*

We would like to thank the Reviewer for pointing this out. *Fam3c* is the official gene symbol for the mouse gene, but as we most often refer to the protein as ILEI we have now replaced Fam3c and ILEI transgenic descriptions with *Fam3c/ILEI* to avoid any misunderstanding.
